# The degradation during recycling of polyamide 6 produced by anionic ring-opening polymerization of ε-caprolactam

**DOI:** 10.1038/s41598-023-44314-0

**Published:** 2023-10-10

**Authors:** Orsolya Viktória Semperger, András Suplicz

**Affiliations:** 1 Department of Polymer Engineering, Faculty of Mechanical Engineering, Budapest University of Technology and Economics, Műegyetem rkp. 3, Budapest, 1111 Hungary; 2 Production Division, Bay Zoltán Nonprofit Ltd. for Applied Research, Kondorfa utca 1, Budapest, 1116 Hungary; 3 MTA-BME Lendület Lightweight Polymer Composites Research Group, Műegyetem rkp. 3, Budapest, 1111 Hungary

**Keywords:** Polymers, Rheology, Characterization and analytical techniques

## Abstract

As the plastics industry continues to grow, the amount of plastic waste is also increasing. The European Union is controlling plastic waste through various regulations, focusing primarily on recyclability. A good alternative to traditional thermoset composites is thermoplastic polyamide 6 composites produced by Thermoplastic Resin Transfer Molding (T-RTM). Polyamide 6 has high strength and is produced by in-situ anionic ring-opening polymerization in T-RTM. Products made with this technology can replace traditional thermoset composites in many areas, which greatly increases recyclability. In this paper, the recyclability of the high molecular weight polyamide 6 matrix material of T-RTM composites is investigated. Products were mechanically recycled and then processed by injection molding. Thermal, mechanical and rheological properties of the samples were compared with the properties of the original product, as well as a general injection molding–grade PA6. Results show that the parts prepared with this innovative technology can be mechanically recycled and reprocessed by injection molding without a processing aid. After reprocessing, a significant reduction in properties is observed due to degradation, but the properties of the resulting product are in good agreement with those of a conventional commercially available injection molding grade PA6 material.

## Introduction

Due to the continuous development of the plastics industry, the annual production of plastics has been increasing for more than 50 years. In 2020, the volume of plastic products produced increased to 368 million tons, an increase of about 2.4% compared to 2018^1^. At the same time, the amount of plastic waste has been steadily increasing as well, reaching 300 million tons per year in recent years, and its disposal is a global problem. To tackle this problem, countries have developed different strategies. The waste management policy of the European Union is one of the most advanced, with the reduction, reuse and recycling of waste in landfills as its main objective^2,3^.

With up to 8–9 million tons of waste generated by the automotive industry each year, these regulations will bring about a major change in this area, supporting new technological developments and eco-innovation. A regulation^4^ was also adopted requiring, among other things, that by 1 January 2015 at the latest, cars should be 95% recyclable by weight. In addition, stricter CO_2_ emission standards for vehicles have been introduced, requiring new passenger cars to have CO_2_ emissions below 95 g/km from 2020 and 75 g/km by 2030. These regulations were also intended to encourage the car industry to innovate technologically, promoting eco-innovation^5,6^. For these reasons, one of the main directions of this research is thermoplastic polymer composite technologies, which can be alternatives to metal components, due to their superior mechanical properties, lightweight and recyclability. Previously, the problem with continuous fiber–reinforced thermoplastic composite manufacturing technologies was that the high viscosity of the thermoplastic matrix material did not allow the sufficient impregnation of the reinforcing fibers, limiting the spread of the technology. The solution could be Thermoplastic Resin Transfer Molding (T-RTM), which is ideally suited for anionic ring-opening polymerization^7–9^. T-RTM has advantages over thermoset composite manufacturing technologies, such as recyclability, lower environmental impact, and the ability to create different layering patterns of reinforcing fabrics within a product for different loads. Furthermore, the short cycle time makes the technology suitable for mass production^10^. Thanks to the low temperatures and pressures used, large, thick and complex geometries can also be produced^10,11^.

Due to their excellent properties, polyamides are widely used engineering polymers. They are semi-crystalline polymers^12–14^. In recent years, the demand for polyamides has reached 1 million tons, most of which has been used in the transport, electronics and construction industries^2^. The anionic ring-opening polymerization process allows the low viscosity monomer of polyamide 6, ε-caprolactam, to surround the fibers completely, without pores, and the final product to be formed as the last step in the polymerization process^15–20^. Moreover, in addition to the material and energy recovery typical with thermoplastic polymers, polyamide 6 also offers the possibility of chemical recycling, as it can be depolymerized to the starting monomer in a cost-effective way^21^.

One of the main ways to recycle polymers is mechanical recycling, whereby the thermoplastic polymer composite is shredded, melted and then injection molded or extruded. The process involves the use of a low-speed cutting or crushing machine to shred larger pieces into smaller pieces and is mostly used by manufacturers of glass fiber–reinforced composites. The resulting materials can be mixed with the original material and re-molded or extruded. The disadvantage of mechanical recycling is that the material degrades during melting and reprocessing, which reduces macromolecular length and molecular weight and this has a major impact on mechanical properties and processability. However, incorrect processing parameters (e.g. too high a temperature) cause more damage than repeated processing at the right temperature. In many cases, recycling is possible without a significant change in properties. Furthermore, due to advanced quality control, thermoplastic polymers can be injection molded or extruded ten times without noticeable molecular degradation if heat stabilizers are added^21–24^.

The aim of this research is to investigate the mechanical recyclability and reprocessability of polyamide 6 products produced by anionic ring-opening polymerization with the use of T-RTM, an innovative technology. As there have been no publications in this area so far, our further aim is to show how the properties of the raw material change during recycling. For this purpose, the products obtained by ring-opening polymerization were mechanically recycled, then injection-molded and their properties were compared to the original product and to a commercial injection molding polyamide 6.

## Materials and methods

### Materials

For the preparation of the test samples, an ε-caprolactam/C10/C20P system was used, which can be applied for the production of PA6 by in-situ polymerization with short cycle times and anionic ring-opening below the melting point (130–170 °C). The starting material was AP- Nylon ε-caprolactam (L. Brüggemann GmbH & Co. KG, Germany), which has a melting point of 69 °C, a density of 0.6–0.7 g/cm^3^ and a viscosity in the liquid state similar to that of water (3–5 mPas). Due to its high moisture absorption capacity, it was stored under vacuum. Sodium caprolactam, with a density of 0.45–0.55 g/cm^3^ and a melting point of 62.2 °C, was used as initiator (Brüggolen C10, L. Brüggemann GmbH & Co. KG, Germany). Like ε-caprolactam, C10 is sensitive to moisture and easily deactivated in the presence of water, therefore it was stored under vacuum as well. Hexamethylene-1,6-dicarbamoyl caprolactam (Brüggolen C20P, L. Brüggemann GmbH & Co. KG, Germany) was used as activator. It has a density of 0.85 g/cm^3^ and a melting point of 70 °C.

For the reference sample, Durethan B30S (Lanxess) polyamide 6 granules were used. This material, which is commonly used in injection molding, allows the production of products with good mechanical properties, such as high impact resistance. It has a melting point of 222 °C and a density of 1.14 g/cm^3^ at room temperature.

### Preparation of samples

**Figure 1:**
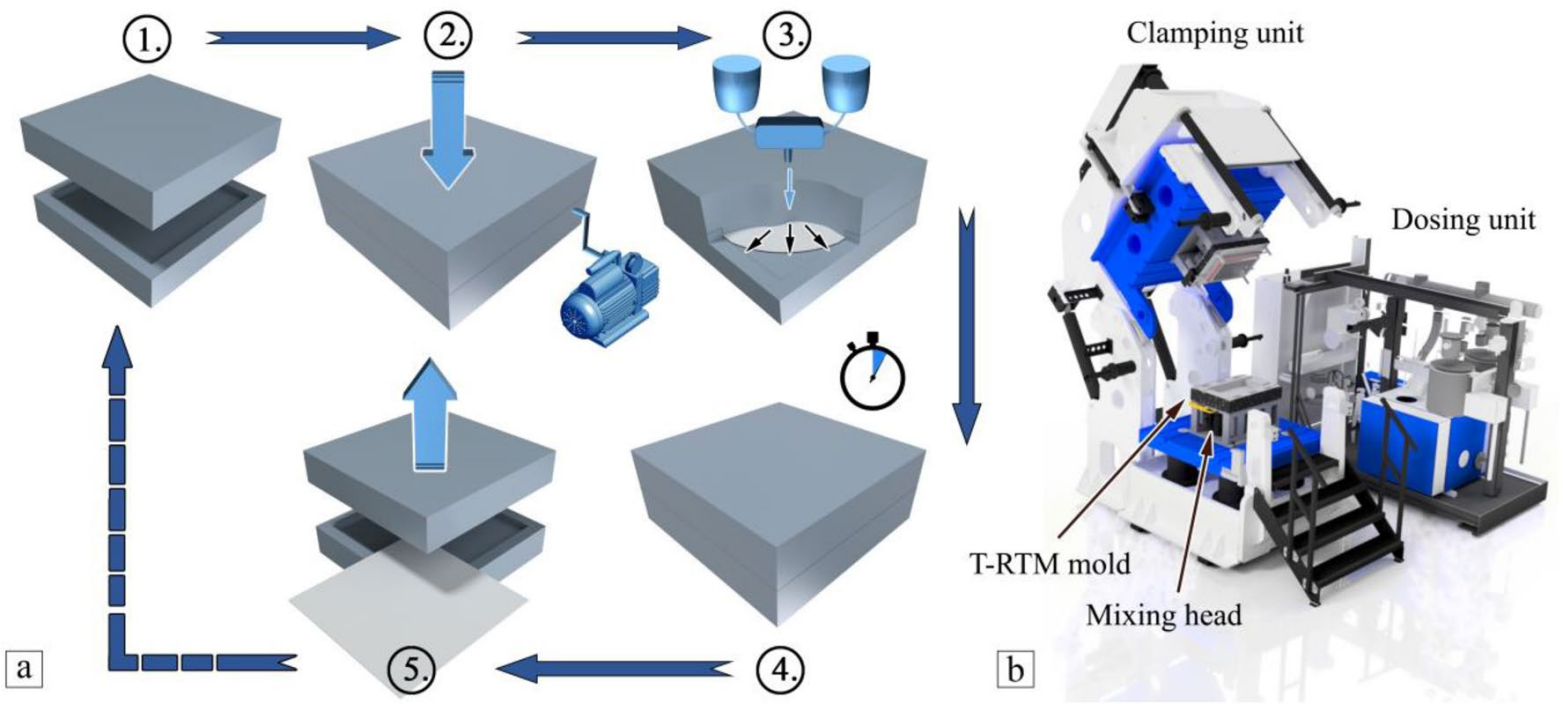
Sample preparation (**a**) with the T-RTM unit (1, mold preparation for start; 2, mold closing and vacuuming; 3, mold filling; 4, polymerization; 5, mold opening and part removal) and the main parts of the T-RTM machine (**b**).

Sheet-like specimens (510 mm × 290 mm × 2 mm) were produced with T-RTM and in-situ polymerization with equipment from KraussMaffei. The equipment consists of dosing units (DU) and a hydraulic press (Fig. 1). The DU is responsible for melting the raw material, storing it in an airtight container, transporting it to the mold carrier press and injecting it into the mold. The unit consists of two DUs, one (DU1) for the base and the other (DU2) for surface coating. The function of the press is to provide clamping force for the mold. The first step in the production process was to measure out the appropriate amount of raw materials—CL-92.5 m/m% (caprolactam), C10-4.5 m/m% (catalyst), C20P-3 m/m% (activator)—and feed them into the DU.

The DU has two tanks for melting CL + C10 and CL + C20P separately. The raw materials were melted in the DUs at 110 °C in an inert, nitrogen atmosphere, followed by vacuum deaeration and the provision of a nitrogen atmosphere. During operation, the melt circulates continuously in a closed system so that contact with air is avoided. Once the raw material is melted, it is conveyed through heated tubes to the forming die mounted on the press, where the material is fed in by means of a mixing head. The heated mixing head (110 °C) ensures that both components are fed separately into the mold (150 °C), and only come into contact with each other immediately after the mixing head, to avoid a premature start of the polymerization process. Proper mixing of the components from the two separate tanks is ensured by the right volumetric flow rate and the design of the mixing head. The hermetically sealed mold is under vacuum, which ensures the best possible filling. After the filling of the mold and the polymerization time (180 s), the finished specimen can be removed. This specimen (T-RTM) was used directly for testing.

**Figure 2:**
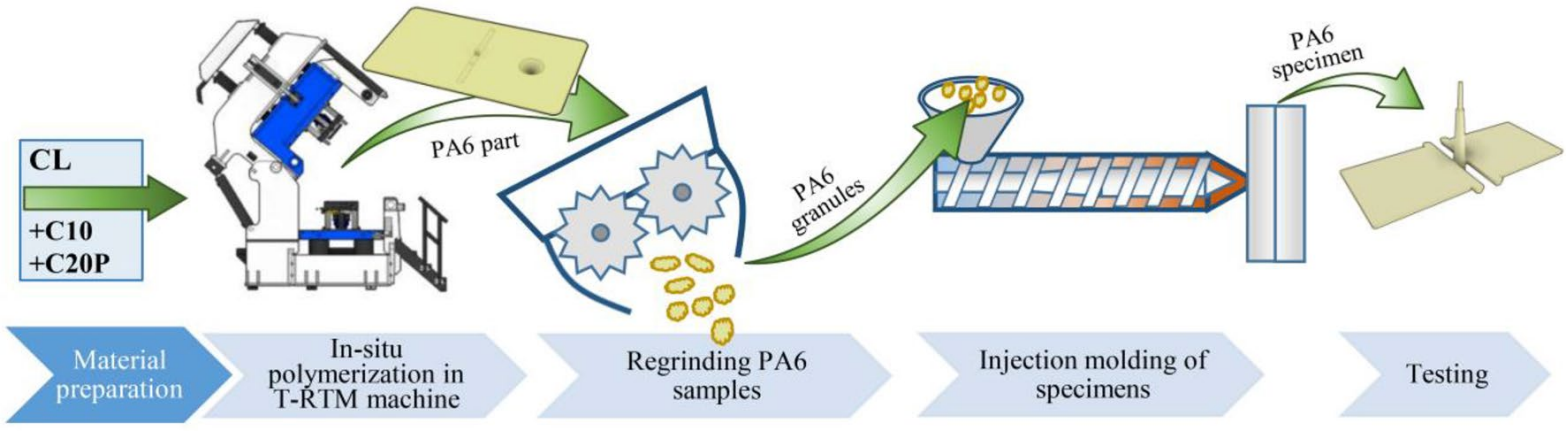
Reprocessing of samples prepared by T-RTM.

From the unreinforced polyamide 6 specimens produced by T-RTM, shredded 5 mm polyamide 6 pellets were produced with an Sb Plastics Machinery GRS202 shredder. From both the T-RTM shredded pellets (T-RTM (re)) and the Durethan B30S polycondensation polyamide 6 pellets (IM grade), 80 × 80×2 mm sheets were produced by injection molding in a two-cavity cold-runner mold at a melt temperature of 250 °C (Fig. 2). The samples were injection molded with an Arburg 270S 400–170 machine. The injection molded specimens were subjected to the same tests as the original T-RTM specimens.

### Characterization of the samples

Samples were conditioned in an exicator at 23 °C for 48 h at 50% relative humidity before testing. The samples for each test were prepared from 2 mm thick 80 × 80 mm injection molded and 510 × 290 × 2 mm T-RTM plates.

The degradation, decomposition and monomer concentration of the samples were investigated by thermogravimetric analysis (TGA Q500, TA Instruments, New Castle, USA). The samples were heated to 400 °C in a nitrogen atmosphere at 10 °C/min. The B30S material was heated to 600 °C.

The crystalline fraction of the 5 mg samples was determined with a DSC Q2000 (TA Instruments, New Castle, USA) machine in a heat-cool-heat cycle, in nitrogen. The measuring range was 25 °C to 250 °C and the heating/cooling rate was 10 °C/min. The crystalline fraction was calculated with Eq. (1):

1 $$X = \frac{{\Delta H_{m} - \Delta H_{cc} }}{{\Delta H_{f} \Delta \left( {1 - \varphi } \right)}}$$

where *ΔH*_*m*_ is the enthalpy of melting, *ΔH*_*cc*_ is the enthalpy of cold crystallization, *ΔH*_*f*_ is the melting enthalpy of a theoretically fully crystalline polymer (188 J/g for PA6^25^) and *φ* is the mass fraction of the filler.

The relative viscosity of the polyamide 6 samples was determined with a PSL Rheotek RPV-1 automatic solution viscometer according to ASTM D4603. The solvent used was a mixture of phenol/1,1,2,2-tetrachloroethane (60%/40%) (Sigma-Aldrich, USA). The measurements were carried out at 30 °C on 0.5 dl/g polymer solutions. The relative viscosity of the samples was determined from the flow time of the solutions and the solvent through the capillary, with Eq. (2):

2 $${\eta }_{rel}=\frac{t}{{t}_{0}}$$

where η_rel_ [–] is relative viscosity, t [s] is the flow time of the polymer solution and t_0_ [s] is the flow time of the pure solvent.

A Bruker Tensor II, Attenuated Total Reflectance Infrared Spectroscopy apparatus (Bruker Optics Inc., USA) was used to analyze the chemical structure of polyamide 6 samples. Spectra were taken between 4000 and 400 cm^-1^; 16 scans were performed.

A Zwick Z020 (Zwick, Ulm, Germany) tensile testing machine was used to test the standard Type 5A specimens (ISO 527–2) under uniaxial tensile loading at constant tensile speed. The machine recorded the tensile force as a function of length change. The dumbbell-shaped specimens were 75 mm long, 12.5 mm wide and 2 mm thick. Grip length was 50 mm and test speed was 5 mm/min. Tensile strength and tensile modulus were calculated.

The bending tests were also carried out on a Zwick Z020 (Zwick, Ulm, Germany) universal tensile testing machine. Specimen size was 40 mm × 25 mm × 2 mm, support spacing was 32 mm and the loading rate was 5 mm/min. The test was performed up to the conventional deflection, which is equal to 1.5 times the specimen thickness (i.e. 3 mm in this case). From the evaluation limit, bending stress and the flexural modulus of elasticity were determined.

The Charpy tests were performed on a Resil Impactor Junior (Ceast, Torino, Italy) tester. For the instrumented impact test, specimens of 80 mm × 10 mm × 2 mm were cut from each sample and a 2 mm deep notch was made on them. The test was performed with a 2 J hammer with a support distance of 62 mm. From the instrumented test results, impact strength and the ductility index (3) were determined.

3 $$DI=\frac{{E}_{\mathrm{Fmax}}}{{E}_{\mathrm{total}}}$$

where *E*_Fmax_ [kJ] is the energy absorbed up to the maximum force and *E*_total_ [kJ] is the energy absorbed up to the first zero transition.

A Ceast SR 50 capillary rheometer (Ceast, Turin, Italy) was used to measure the melt viscosity of the materials. 2 capillaries of different lengths (5 and 20 mm) and the same diameter (1 mm) were inserted into the apparatus. The materials were measured at 3 different temperatures (240 °C, 260 °C and 280 °C) and at 7 different shear rates (100–10 000 1/s). The corrections necessary for evaluation (Bagley and Rabinowitch corrections) were automatically performed by the software of the instrument using the values measured with the two capillaries.

The Cross-WLF model was used to model the effect of temperature and shear rate on viscosity. The model can be described as follow (4):

4 $$\eta \left(\dot{\gamma }\right)=\frac{{\eta }_{0}}{1+{\left(\frac{{\eta }_{0}\cdot \dot{\gamma }}{{\tau }^{*}}\right)}^{1-n}}$$

where *η* [Pas] is the viscosity of the polymer melt, *η*_0_ [Pas] is the zero shear-rate viscosity, *γ* [1/s] is the shear rate, τ* [Pa] is the critical shear stress at the transition from the Newtonian plateau level to shear thinning, and *n* is the power law index. The temperature-dependent zero shear viscosity is given by the WLF equation (5):

5 $${\eta }_{0}(T)={D}_{1}\cdot exp\left[-\frac{{A}_{1}\cdot \left(T-{T}^{*}\right)}{{A}_{2}+\left(T-{T}^{*}\right)}\right]$$

where *T* [K] is the temperature, T* [K] is the glass transition temperature (320 K ^25^), and *D*_1_ [Pas], *A*_1_ [−] and *A*_2_ [K] are data-fitted coefficients ^26^.

## Results and discussion

### Monomer concentration of the samples

In the production of PA6 by anionic in-situ polymerization, attention should be paid to residual monomer content, as non-polymerizable monomers and oligomers can modify the properties of the final product as plasticizers. The maximum non-reacting monomer content in the product is 4–5%, above which it should be removed.

**Figure 3:**
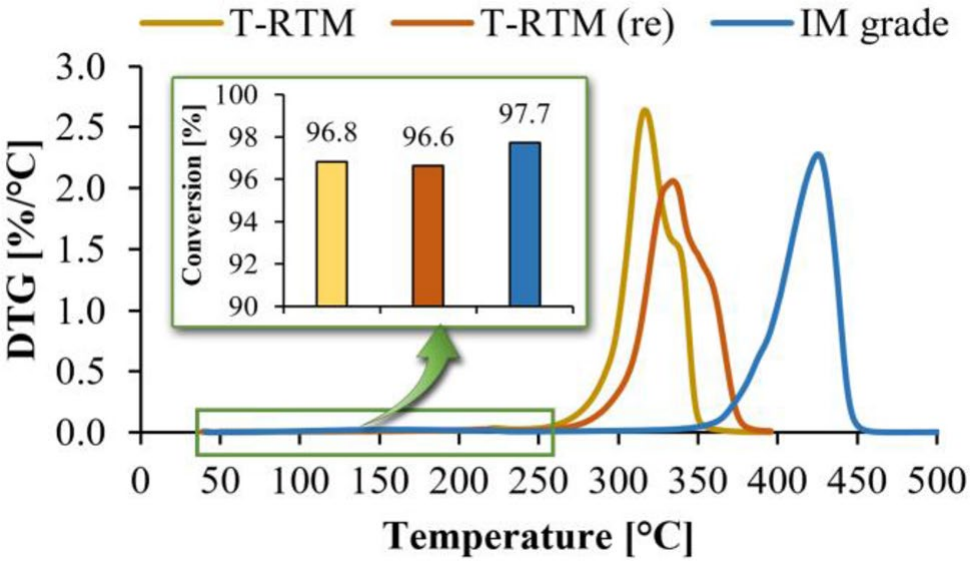
DTG curves of samples prepared with different technologies; Residual monomer content of the different samples.

The residual monomer content in each sample was determined by TGA based on the work of Samet Kurt et al.^27^. Minor changes in the initial stages of the TGA and DTG curves indicate the removal of absorbed water (up to about 80–100 °C) and residual monomer and oligomer (between about 100 °C and 240 °C) from the sample (Fig. 3). The significant weight loss of PA6 samples produced by T-RTM starts at ~ 260 °C, so we used the percentage weight at this temperature to determine conversion by compensating for the initial moisture content of the sample (~ 1.4%). The results indicate that the conversion efficiency of the sample was 96.8%. The reprocessed T-RTM test specimens had a similar degree of polymerization compared to the starting material (96.6%). The sample injection molded from polycondensation granules also showed similar conversion (97.7%, see Fig. 3), but the decomposition temperature of these samples starts around 350 °C. The sample produced by T-RTM had the lowest decomposition temperature. These different decomposition temperatures may be due to the different material structures of the samples and the absence of a stabilizer. The samples can be processed at a maximum melt temperature of 250 °C, above which significant degradation occurs.

### Effect of technology on crystallinity

The semi-crystalline structure of PA6 consists of ordered crystalline molecular chains and disordered amorphous domains. While the former improves strength and stiffness, the latter provides toughness and elongation.

**Figure 4:**
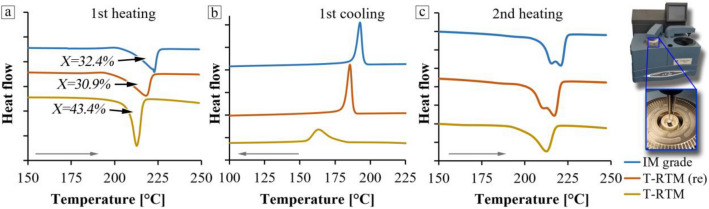
DSC curves of the PA6 samples (**a**, 1st heating; **b**, 1st cooling; **c**, 2nd heating).

The crystalline fraction of the samples was determined from the curves obtained by DSC (Fig. 4). Based on the first heating curves, the T-RTM samples had a higher crystalline fraction (43.4%) than the re-injection-molded and injection-molded samples. Based on the first heating curves, the injection-molded (IM grade) samples had a crystalline fraction of 32.39%, while the re-injected material (T-RTM (re)) had a crystalline fraction of 30.85%. The decrease in the crystalline fraction due to reprocessing may be caused by the different nature of the production technologies. The first heating curves show that the original T-RTM samples have a narrower crystal melting range than the injection-molded samples, which may be due to the narrower, more uniform distribution of the crystal lamella size (Fig. 4).

Furthermore, the shift of the crystallization peak due to the reprocessing, i.e. the increase of the crystallization temperature from 163.13 to 185.48 °C^28^, can be seen in the cooling curves of the T-RTM product. In addition, there is a decrease in the width of the exothermic peak, all of which indicate an increase in the crystallization rate. Since the starting material was not changed, only reprocessed, it is likely that this is related to the reduction of the length of the molecular chains, their degradation. Samples prepared by T-RTM have longer molecular chains, which are therefore less mobile, so crystallization starts later and occurs more slowly than in the case of injection-molded samples, which are likely to have shorter chains.

**Figure 5:**
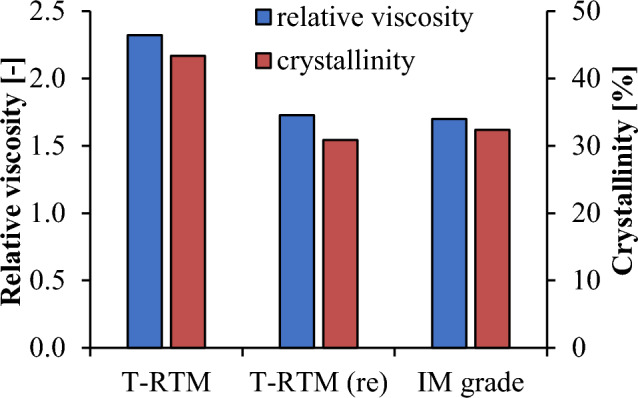
Relative viscosity and crystallinity of the PA6 samples.

The change in the length of the molecular chains can be well characterized by the change in the viscosity of the polymer solutions. Figure 5 shows the relative viscosity of the PA6 samples. The specimen produced by T-RTM has the highest relative viscosity (2.3). In comparison, the recycled and IM grade samples have a relative viscosity more than 25% lower, 1.73 and 1.7, respectively. This indicates that mechanical recycling has significantly reduced the molecular weight of anionically polymerized PA6. In addition to molecular weight, crystalline fraction is also significantly reduced, which can lead to a deterioration of mechanical properties. Furthermore, the molecular weight and the crystalline fraction of the recycled and IM grade material are almost the same (Fig. 5), thus similar mechanical properties can be expected.

During the DSC analysis, in the second heating cycle (Fig. 4), there is a smaller peak next to the main endothermic peak, due to reprocessing. This may be because two stable crystal structures (α and γ) can be distinguished in polyamide 6. The α structure has higher thermal and mechanical stability due to its more regular crystalline structure, because the chains are in a stretched state. It has a melting temperature of 222 °C, while the γ structure has a melting temperature of 215 °C. In the latter, the chains are twisted, which also changes the angle of the bonds between them, resulting in less stability^29^. Furthermore, as the number of recycling cycles increases, the average molecular weight decreases, but this secondary endothermic peak increases steadily; this way they show proportionality with each other.

### Structural characterization

The structure and structural changes of polyamide 6 samples were analyzed by ATR-FTIR. Since the –CH_2_– content is relatively unchanged in polyamide 6, the spectra were normalized to these peaks^30^.

**Figure 6:**
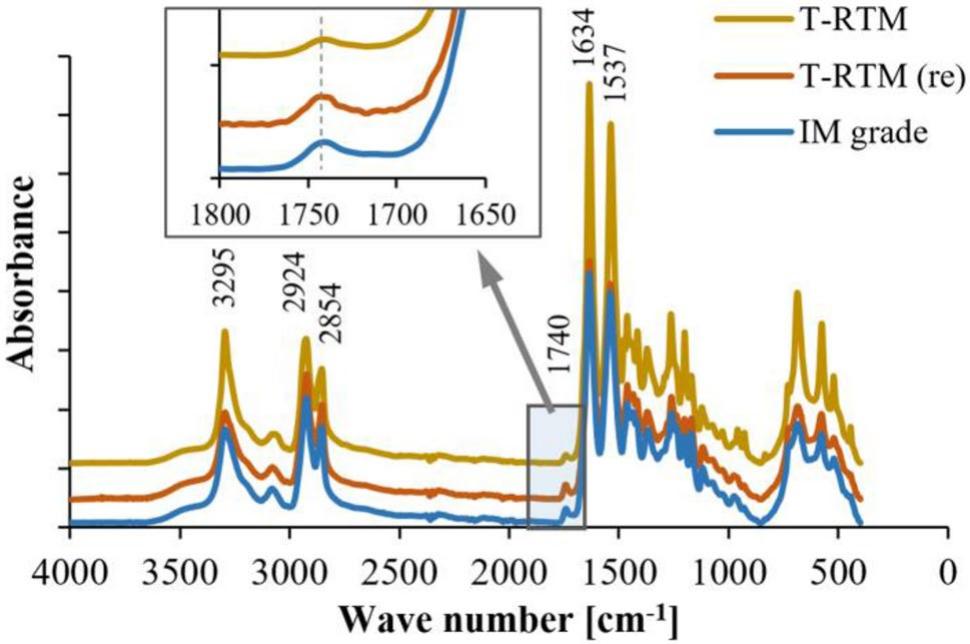
ATR-FTIR analysis of the PA6 samples.

First, the main bonds characterizing polyamides were determined. The peak at 3295 cm^−1^ is attributed to the hydrogen-bonded N–H stretching. Bands around 2924 and 2854 cm^−1^ are assigned to methylene (C–H) stretching or tension vibration. The peaks around 1634 and 1537 cm^−1^ correspond to amide I (C = O) and amide II (N–H and C–N combination), respectively^31–33^. As Fig. 6 shows, no new absorption peak appears on the spectra of the reprocessed T-RTM sample. Also, no significant changes can be observed in the main peaks of the spectra. Similarly Mondragon et al.^31^, a change was also observed at 1740 cm^-1^. This peak was five times higher after recycling. It can be associated with the stretching vibration of the carboxyl group (-COOH), probably formed due to the oxidation of peptide links of PAs during recycling. This may also prove the shortening of the chains assumed from the results of the solution viscosity measurement.

### Mechanical characterization

#### Tensile testing

**Figure 7:**
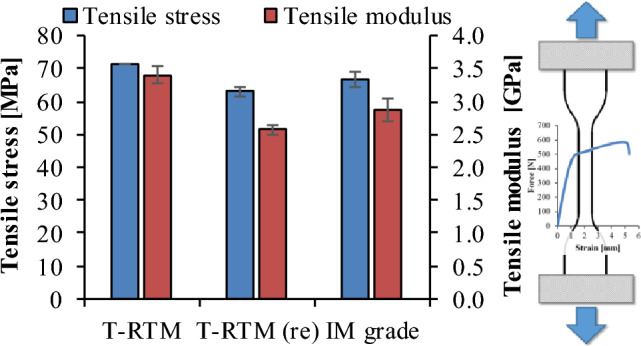
Tensile strength and tensile modulus of elasticity.

Samples produced by T-RTM had higher strength than those re-injection-molded or injection-molded from polycondensation PA. While the former had a tensile strength of 69 MPa and a tensile modulus of elasticity of 3.3 GPa, the specimen injection molded from granules had a tensile strength of approximately 66 MPa and a tensile modulus of elasticity of 2.8 GPa (Fig. 7). The reason for this is the higher crystalline fraction of the specimens produced by T-RTM. The strength and stiffness of the re-molded PA6 specimens were only slightly inferior to those of the injection-molded specimens from polycondensation PA.

**Figure 8:**
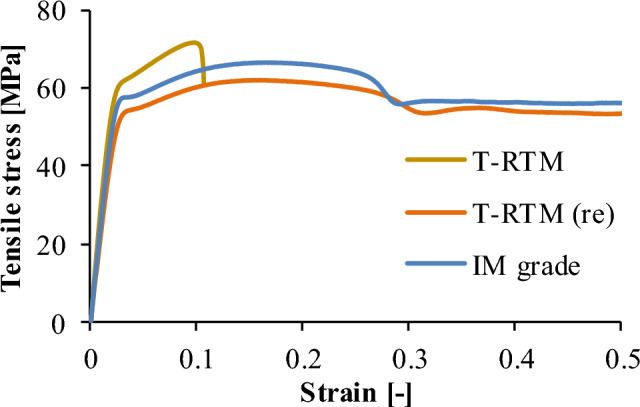
Tensile stress-strain curves of the samples.

The tensile curves (Fig. 8) show that IM grade and recycled samples exhibited significant neck formation during tensile testing and only broke after more than 50% elongation. The nature of the two curves is similar, with identical behavior. The IM grade material has 50.3% (± 3.8%) elongation at break, and the recycled sample has 85.5% (± 9.19%). In comparison, the maximum elongation of the samples prepared by anionic polymerization is much lower: 10.8% (± 1.1%). This different character is also due to the different crystal fraction of polyamides.

#### Bending

**Figure 9:**
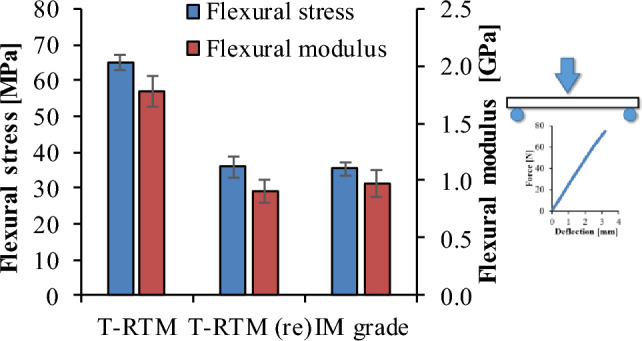
Bending stress and flexural modulus of elasticity.

Stiffness and strength were also greatly improved by the crystalline parts. The re-injection-molded specimens had the lowest crystalline fraction (30.85%) and ultimate flexural stress (35.8 MPa), while for the T-RTM specimens these values were 43% and 65 MPa, respectively. The in-situ polymerization products have higher stiffness than the injection-molded products. In addition to the highest flexural stress, the T-RTM samples also had the highest modulus of 1.88 GPa. Nevertheless, the T-RTM re-injection-molded specimen and polycondensation PA have similarly good mechanical properties (Fig. 9).

#### Charpy impact testing

**Figure 10:**
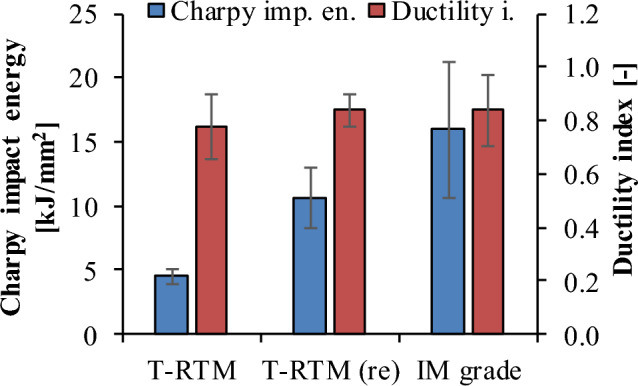
Impact work and ductility index.

The Charpy impact strength of the T-RTM samples was 4.5 kJ/m^2^. The approximately 13% less crystalline fraction in the re-injection-molded sample resulted in an increase of about 6 kJ/m^2^ in impact work (Fig. 10). The ductility index (DI) of the samples was also calculated, which showed values close to 1 for all of them. DI values close to 1 indicate brittle fracture, while lower DI indicates tougher fracture. The injection-molded specimens had a higher average DI, i.e. they were more brittle than the specimens produced by T-RTM. However, we cannot show a significant difference between the measurement results due to the large standard deviations. In the case of the T-RTM samples, the brittle behavior is due to the notch and the high crystalline fraction. In the case of injection-molded samples, this can also be caused by the notch and shorter molecular chains.

### Rheology

**Figure 11:**
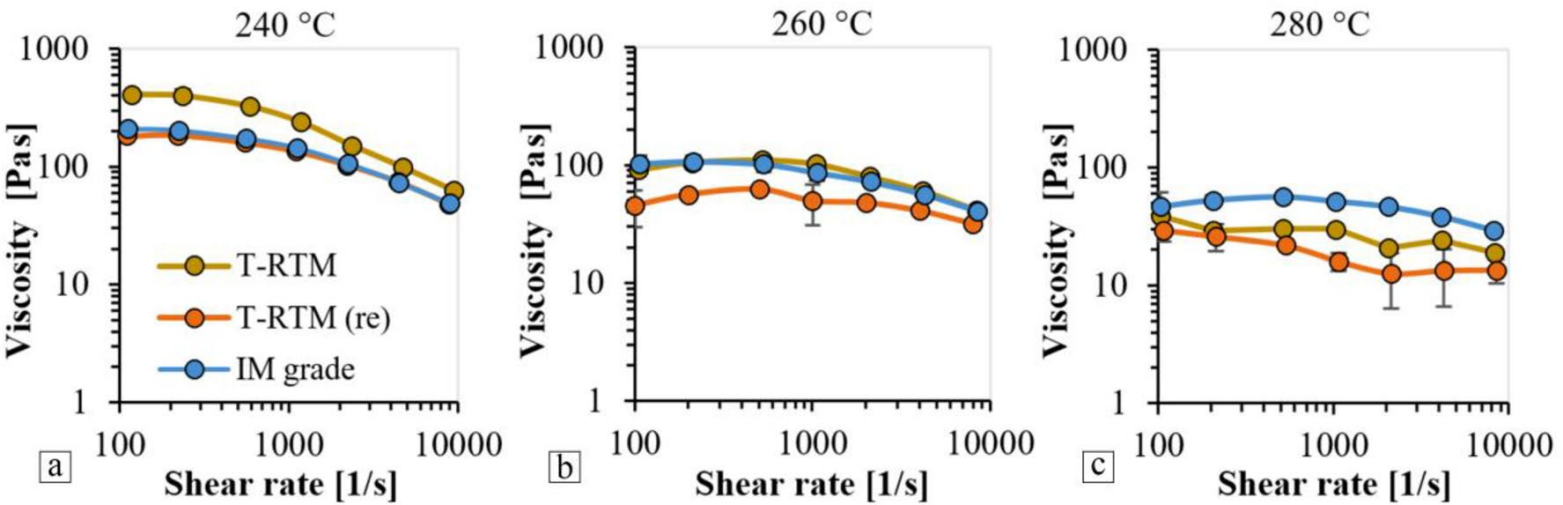
Viscosity curves at (**a**) 240 °C, (**b**) 260 °C and (**c**) 280 °C.

Figure 11 shows the viscosity curves of specimens produced with different technologies at 240, 260 and 280 °C. At a test temperature of 240 °C, the viscosity curves of the samples produced with different technologies are different from each other. The samples produced by T-RTM had higher viscosity (about 400 Pas at a shear rate of 100 1/s), which indicate higher molecular weight—as the molecular weight of a given material increases, its viscosity also increases. As shown previously, the properties of the recycled T-RTM samples and the injection molding grade material are very similar, and their viscosity curves show good agreement as a function of shear rate at 240 °C (from 200 to 50 Pas between 100 and 10,000 1/s shear rate). The lower viscosity of the reused material compared to the original T-RTM product was due to the mechanical and thermal effects (grinding and melting) during reprocessing, which resulted in a reduction in molecular weight. The difference between the viscosity of the two materials at a shear rate of 100 1/s is 230 Pas. As shear rate increases, the viscosity of all three materials decreases, and the difference between them also tends to decrease.

**Figure 12:**
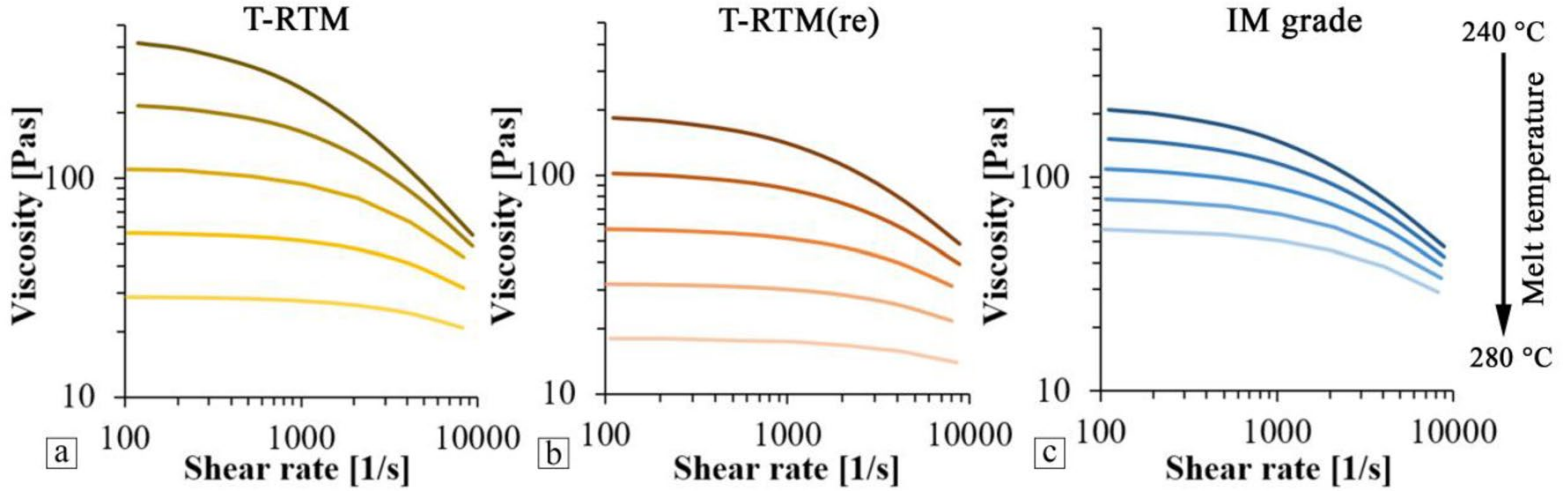
Cross-WLF model of the viscosity of the polyamide samples between 240 and 280 °C (**a**, T-RTM; **b**, T-RTM(re), **c**, IM grade samples).

The temperature- and shear-dependent viscosity of the polyamide samples were described by the Cross-WLF model between 240  and 280 °C (Fig. 12). The parameters of the model are presented in Table 1. The correlation (R^2^) between the model and the measured results was above 0.995 in all cases. The results show that the temperature dependence of the viscosity of the samples prepared by anionic polymerization and T-RTM is more significant than that of polycondensation PA. While the IM grade material becomes more stable with increasing melt temperature, the viscosity of T-RTM and T-RTM(re) materials shows a significant decrease. One of the reasons is that the difference between the decomposition temperatures of the samples is close to 100 °C. The TGA results showed that the T-RTM samples started to decompose at around 260 °C, with significant degradation occurring at higher temperatures. However, as the temperature increased, the viscosity of both the reprocessed and the T-RTM materials decreased more and more significantly. This may be because at higher temperatures; the chains started to break and degrade. At 280 °C, the viscosity of the polymerized materials is almost the same, about 20–30 Pas at low shear rates. Consequently, the maximum reprocessing temperature for these T-RTM samples is about 250 °C. Therefore, during the recycling of products produced by T-RTM, a stabilizer has to be added to ensure proper processability.

**Table 1:** Parameters of Cross-WLF model.

Material	n	τ*	D_1_	T*	A_1_	A_2_
	[–]	[Pa]	[Pas]	[K]	[–]	[K]
T-RTM	0.05	596,596	3.24E + 11	320	66.6	438
T-RTM(re)	0.098	485,183	1.19E + 11	320	47.4	260.5
IM grade	0.077	481,959	1.10E + 15	320	39.1	64.9

## Conclusion

The goal was to investigate the mechanical recyclability of polyamide 6 produced by in-situ polymerization, by T-RTM. We compared the thermal, mechanical and rheological properties of the starting and recycled polyamide 6 and polycondensation polyamide 6 (injection molding grade). Determining the residual monomer content proved to be very important, as its increase negatively affects mechanical properties. The results indicate that industrial PA6 has higher conversion—they have a lower residual monomer content. However, in-situ polymerization samples also have an acceptable monomer content of 2–2.5%. An increase in the crystalline fraction can result in increased strength and modulus and decreased impact strength. The tests showed that in-situ polymerized PA6 samples had a crystalline fraction more than 10% higher than PA6 samples injection molded from granules or pellets. The solution viscosity measurements showed that the anionically polymerized samples had the highest molecular weight, which can also have a significant effect on mechanical properties.

Recycling reduced molecular weight, which was thus close to the molecular weight of the IM grade material. This chain scission was also confirmed by ATR-FTIR. Tensile and flexural tests show that in-situ polymerization can produce products with higher strength than injection molding. Although the properties of recycled PA6 deteriorated compared to the starting material, its properties were almost the same as in the case of injection-molded PA6. The results of the Charpy impact test showed that the recycled samples exhibited higher impact strength due to the larger amorphous domains compared to the PA6 samples produced by T-RTM. Viscosity measurements demonstrated that processing at temperatures above 260 °C lead to the degradation of the material, which also cause a significant decrease in molecular weight and hence in viscosity. It was found that polyamide 6 products prepared from ε-caprolactam by anionic ring-opening polymerization with T-RTM technology can be easily recycled and reprocessed by injection molding without a processing aid. If melt temperature during reprocessing is kept below 250 °C, which is the beginning of the decomposition of anionically polymerized PA6, the properties of the resulting product are in good agreement with those of a conventional commercially available injection molding–grade PA6 material.

## Data Availability

The data that support the findings of this study are available from the corresponding author upon reasonable request.
